# Highly Efficient 2D/3D Mixed-Dimensional Cs_2_PbI_2_Cl_2_/CsPbI_2.5_Br_0.5_ Perovskite Solar Cells Prepared by Methanol/Isopropanol Treatment

**DOI:** 10.3390/nano13071239

**Published:** 2023-03-31

**Authors:** Bicui Li, Shujie Yang, Huifang Han, Huijing Liu, Hang Zhao, Zhenzhen Li, Jia Xu, Jianxi Yao

**Affiliations:** 1 State Key Laboratory of Alternate Electrical Power System with Renewable Energy Sources, North China Electric Power University, Beijing 102206, China; 2 Beijing Key Laboratory of Energy Safety and Clean Utilization, North China Electric Power University, Beijing 102206, China; 3 College of Metallurgy and Energy, North China University of Science and Technology, Tangshan 063210, China

**Keywords:** mixed-dimensional perovskite, all-inorganic perovskite, Cs_2_PbI_2_Cl_2_

## Abstract

All-inorganic perovskite solar cells are attractive photovoltaic devices because of their excellent optoelectronic performance and thermal stability. Unfortunately, the currently used efficient inorganic perovskite materials can spontaneously transform into undesirable phases without light-absorption properties. Studies have been carried out to stabilize all-inorganic perovskite by mixing low-dimensional perovskite. Compared with organic two-dimensional (2D) perovskite, inorganic 2D Cs_2_PbI_2_Cl_2_ shows superior thermal stability. Our group has successfully fabricated 2D/3D mixed-dimensional Cs_2_PbI_2_Cl_2_/CsPbI_2.5_Br_0.5_ films with increasing phase stability. The high boiling point of dimethyl sulfoxide (DMSO) makes it a preferred solvent in the preparation of Cs_2_PbI_2_Cl_2_/CsPbI_2.5_Br_0.5_ inorganic perovskite. When the perovskite films are prepared by the one-step solution method, it is difficult to evaporate the residual solvent molecules from the prefabricated films, resulting in films with rough surface morphology and high defect density. This study used the rapid precipitation method to control the formation of perovskite by treating it with methanol/isopropanol (MT/IPA) mixed solvent to produce densely packed, smooth, and high-crystallized perovskite films. The bulk defects and the carrier transport barrier of the interface were effectively reduced, which decreased the recombination of the carriers in the device. As a result, this effectively improved photoelectric performance. Through treatment with MT/IPA, the photoelectric conversion efficiency (PCE) of solar cells prepared in the N_2_ atmosphere increased from 13.44% to 14.10%, and the PCE of the device prepared in the air increased from 3.52% to 8.91%.

## 1. Introduction

Organic–inorganic hybrid perovskites exhibiting attractive optoelectronic performance characteristics, such as significant light extinction, excellent defect tolerance, and superior charge transport, are among the most favorable photovoltaic materials [1,2,3]. These hybrid perovskite solar cells’ (PSCs) certified PCE has reached 25.7% [4,5]. Despite their extraordinary achievements, their stability remains a major challenge for commercialization. Considering the decomposable property of CH_3_NH_3_^+^ (MA^+^) and CH(NH_2_)_2_^+^ (FA^+^), hybrid perovskites tend to be unstable at high temperatures, generally suffering from the issue of thermal instability [6,7,8]. Replacing the organic cation with inorganic Cs^+^ is regarded as one effective approach to increase thermal stability [9]. The tolerance temperature of inorganic cesium-based perovskite can be up to 400 °C [10]. Nowadays, many studies have been devoted to obtaining efficient and stable inorganic PSCs, and the PCE has been increased up to 21.35% [11].

However, the ideal cubic phase cesium-based inorganic perovskite can spontaneously transform into a non-perovskite phase, which is harmful to light absorption [12]. Many studies were carried out to stabilize the phase of cesium-based inorganic perovskite for photovoltaic applications, such as additive engineering [13,14,15], compositional optimization [16,17,18,19], and dimensional engineering [20,21]. Because of the excellent stable crystal structure, the lower-dimensional perovskite can effectively stabilize the perovskite black phase by forming a 1D/3D or 2D/3D mixed-dimensional structure [22,23].

Zhang and co-workers [24] introduced the ethylenediamine (EDA) cation to form the 2D EDAPbI_4_ perovskite, which significantly stabilized the phase of α-CsPbI_3_ crystallites and passivated the surface defects. Jiang and co-workers [25] formed quasi-2D perovskites by introducing phenylethylammonium (PEA) into inorganic perovskite, and this suppressed the harmful phase transition, resulting in a PCE of 12.4%. However, the mixed-dimensional structure is mainly based on organic 2D perovskite, which is accompanied by a decrease in thermal stability. In contrast, 2D Cs_2_PbI_2_Cl_2_ exhibits excellent thermodynamic structure stability [26]. Kanatzidis and co-workers [26] proved that Cs_2_PbI_2_Cl_2_ is suitable for solar cell absorption materials owing to its wide band gap of 3.04 eV, strong ultraviolet (UV) response, and long-term stability. Xu and co-workers [27] made a theoretical prediction that 2D Cs_2_PbI_2_Cl_2_ displays ultrahigh carrier mobility of 9.39 × 10^3^ cm^2^/V/s and confirmed its very promising photoelectric performance. Our group [28] successfully fabricated high-quality 2D/3D mixed-dimensional Cs_2_PbI_2_Cl_2_/CsPbI_2.5_Br_0.5_ films, which significantly improved the phase stability, and achieved a considerable increase in efficiency of 15.09%. These results show the potential application prospects of the mixed-dimensional Cs_2_PbI_2_Cl_2_/CsPbI_2.5_Br_0.5_ perovskite structure.

As we know, DMSO has satisfactory halide coordination ability and solubility, so it is the preferred solvent for preparing Cs_2_PbI_2_Cl_2_/CsPbI_2.5_B_r0.5_ perovskite. However, its high boiling point (189 °C) [29] makes the residual DMSO difficult to evaporate from the films during the one-step solution method, resulting in rough surface morphology and tending to create defects. Due to the resulting defect density, which is considerable, a large number of carrier recombination losses limits the performance of the device [30,31]. Therefore, it is required to adjust the crystallization kinetics to improve the quality of the Cs_2_PbI_2_Cl_2_/CsPbI_2.5_Br_0.5_ films. In reference to the research on antisolvent strategy, the use of an antisolvent with high DMSO solubility and a low boiling point is an effective strategy to remove residual solvent molecules from the prefabricated film and form high-quality perovskite film [32].

Here, a mixed solution of methanol (MT) and isopropanol (IPA), which has a weak electron donor ability [33], low boiling point, and poor solubility with halides [34], was used to assist the preparation of Cs_2_PbI_2_Cl_2_/CsPbI_2.5_Br_0.5_ perovskite films. By inducing rapid nucleation and crystallization of the perovskite, the MT/IPA mixed solution can improve the morphology of the films, resulting in densely packed, smooth, and high-crystallized perovskite films. The bulk defects and the carrier transmission barrier of the interface could be obviously reduced, which is valuable for decreasing the recombination of the carriers in the device. As a result, the photoelectric performance is improved, and the PCE of Cs_2_PbI_2_Cl_2_/CsPbI_2.5_Br_0.5_ solar cell devices increases from 13.44% to 14.10%. This method can also be applied to the preparation in the air.

## 2. Materials and Methods

### 2.1. Precursor Material Preparation

Lead (II) diiodide (PbI_2_) and cesium halide (e.g., CsI, CsBr, and CsCl) were purchased from Xi’an Polymer Light. Methanol (MT), isopropanol (IPA), and methyl sulfoxide (DMSO) were purchased from Alfa Aesar. For the 3D precursor solution (CsPbI_2.5_Br_0.5_), 0.5 M CsBr, 0.5 M CsI, and 1.0 M PbI_2_ were dissolved in DMSO. For the 2D/3D precursor solution (Cs_2_PbI_2_Cl_2_/CsPbI_2.5_Br_0.5_), 0.5 M CsBr, 0.5 M CsI, 0.15 M CsCl, and 1.075 M PbI_2_ were dissolved in DMSO. The mixed solution of MT/IPA with different volume ratios is prepared by mixing MT to IPA with a volume ratio of x%. The resulting solution is referred to as “x %-MT/IPA”.

### 2.2. Perovskite Solar Cell Fabrication

The TiO_2_ compact layer was prepared on the substrates using the spraying method. The substrates were pre-heated to 90 °C, and then perovskite was spin coated at a rate of 3000 rpm for 50 s, followed by annealing for 10 min at 325 °C. For the MT/IPA treatment, the mixed solution of MT/IPA was dropped about 15 s before the end of the spin coating. After that, the 2,2′,7,7′-Tetrakis(N,N-p-dimethoxyphenylamino)-9,9′-spirobifluorene (spiro-OMeTAD) was spin coated at a rate of 4000 rpm for 20 s. Thermal evaporation was then used to deposit 65 nm of gold (Au) to finalize the fabrication of the solar cells with an active area of 0.12 cm^2^.

### 2.3. Characterization

An X-ray diffractometer (XRD, SmartLab, Rigaku, Tokyo, Japan) was used to measure the X-ray diffraction (XRD) spectra. The diffraction angles between 5° and 50° were scanned at a speed of 10° per min. An SU8010 scanning electron microscope (SEM) (Hitachi, Tokyo, Japan) was used to obtain the SEM images. Atomic force microscope (AFM) images were recorded by Agilent5500 AFM (Agilent, Santa Clara, CA, USA). Ultraviolet-visible (UV-vis) spectra were recorded by a UV-vis spectrophotometer (UV2450, Shimadzu, Kyoto, Japan). The steady-state photoluminescence (PL) spectra and the time-resolved photoluminescence (TRPL) spectra were recorded by a spectrophotometer (F900, Edinburgh Instruments, Livingston, UK). Films prepared for PL and TRPL were deposited on glass without conducting layers. Electrochemical impedance spectroscopy (EIS) was obtained by the electrochemical workstation (Zahner, Kansas City, MO, USA). A quantum efficiency measurement system (Enli Technology, Kaohsiung, Taiwan) was used to measure the incident photon-to-electron conversion efficiency (IPCE). A source meter (Keithley 2400) with a sunlight simulator (Xes-300T1, SANEI Electric, Tokyo, Japan) was used to measure the current density–voltage (*J–V*) curves and space charge limited current (SCLC). For SCLC measurement, the dual-electron transport layer devices were prepared by replacing the hole transport layer (HTL) with an electron transport material, [6,6]-phenyl C_61_ butyric acid methyl ester (PCBM).

## 3. Results and Discussion

**Figure 1:**
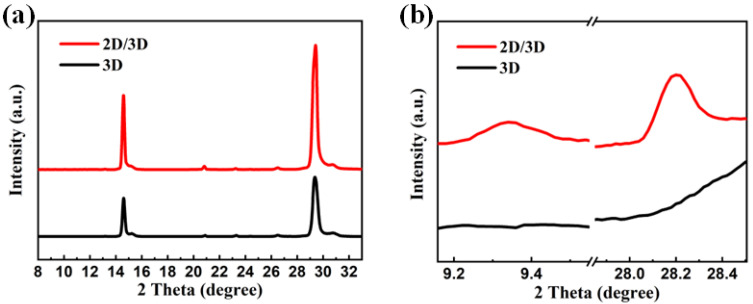
(**a**) Global XRD spectra and (**b**) local magnified XRD spectra of 3D and 2D/3D perovskite films.

Firstly, CsPbI_2.5_Br_0.5_ (3D) and Cs_2_PbI_2_Cl_2_/CsPbI_2.5_Br_0.5_ (2D/3D) perovskite films were prepared using the traditional one-step solution method. In order to form the 2D/3D films, the precursor solution for 2D/3D perovskite was prepared by adding 0.075 mol% CsCl and PbI_2_ to a DMSO solution containing 1 M CsPbI_2.5_Br_0.5_ at a molar ratio of 2:1 [28]. XRD spectra interpretation was carried out in order to demonstrate the production of 2D Cs_2_PbI_2_Cl_2_ in the film and successfully generate mixed-dimension perovskite, as shown in Figure 1a. Around 14.6° and 29.3°, there are two significant diffraction peaks that are apparent in both 3D and 2D/3D films, assigned to (100) and (200) crystal planes of the cubic CsPbI_2.5_Br_0.5_. No visible shift in the diffraction peaks assigned to cubic phases is observed in the 2D/3D film. Rather, the peaks are exactly the same as those in the 3D film. Furthermore, the XRD spectra have been locally magnified around 9.3° and 28.2°. The indexed tiny diffraction peaks that are hard to spot are the (002) and (006) crystal planes of Cs_2_PbI_2_Cl_2_ appear in the 2D/3D films, which indicates the formation of 2D Cs_2_PbI_2_Cl_2_ phase. Moreover, accompanied by the formation of the Cs_2_PbI_2_Cl_2_, there was an apparent increase in the (100) and (200) peaks of the CsPbI_2.5_Br_0.5_. This indicates that the introduction of Cs_2_PbI_2_Cl_2_ results in a significant enhancement in the crystallinity of 2D/3D film.

In order to investigate the effect that the mixed solution of methanol/isopropanol (MT/IPA) has on films that have not been annealed, a measurement of the light absorption was carried out. Figure S1 (Supplementary Materials) shows the UV-vis spectra of varying volume ratios of MT/IPA-treated unannealed films. When the volume ratio of MT/IPA increased, so did the absorption intensity of the unannealed films, indicating that MT promotes the oligomer to transform into the perovskite structure. The devices with the typical planar structure were fabricated in order to demonstrate how the MT/IPA-treated method affects the performance of devices, and to explore an optimal volume ratio of MT/IPA that maximizes device efficiency. The *J–V* curves of the devices prepared by these films are shown in Figure S2 (Supplementary Materials). The device that was treated with an MT/IPA volume ratio of 50% had the best PCE result: 14.10%. Therefore, the 50% MT/IPA-treated devices were further studied to clarify the effect of treatment with MT/IPA mixed solution.

**Figure 2:**
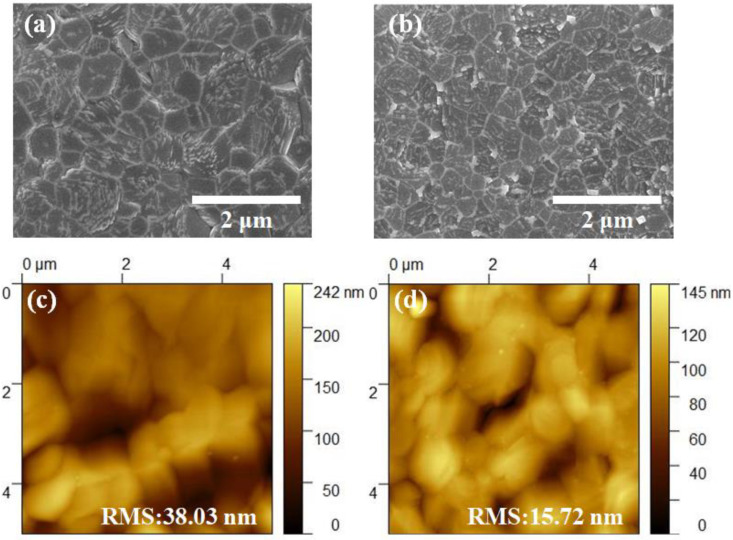
SEM images of (**a**) untreated 2D/3D perovskite films and (**b**) MT/IPA-treated 2D/3D perovskite films; AFM images of (**c**) untreated 2D/3D perovskite films and (**d**) MT/IPA-treated 2D/3D perovskite films.

Investigations were carried out in order to further explore the differences that occurred in the morphology of the 2D/3D films after being treated with MT/IPA. Figure 2a,b are the SEM images. These images demonstrate that the boundaries of grains in untreated film are apparent, and that there are large gaps between the grains. In contrast, the grains in MT/IPA-treated film are denser, which can facilitate carrier transport in the film. Moreover, it was discovered that the average grain size of the untreated film was about 1000 nm, while the grains in films that were treated with MT/IPA were reduced to about 550 nm. It has been identified that the decrease of the perovskite crystal size can bring about the reduction of surface Gibbs free energy, which is helpful in improving stability [35]. The AFM images of both untreated and MT/IPA-treated films are shown in Figure 2c,d. The untreated film has a root means square (RMS) roughness of 38.03 nm, whereas the RMS of the MT/IPA-treated film is only 15.72 nm. The roughness of the film has been greatly reduced by MT/IPA treatment. The high boiling point of DMSO and its strong coordination with the precursor make it difficult for it to evaporate uniformly during annealing. However, the MT/IPA solution causes the rapid removal of DMSO from the precursor solution, which means that the saturation of the solution increases rapidly, resulting in uniform perovskite nucleation. This ensures that DMSO can evaporate completely during the subsequent annealing process, allowing the grains to grow at a uniform speed and leaving a smooth surface. Owing to the smooth surface, which is valuable for interface contact, carrier transport effectively improves. Furthermore, the dense grain surface can reduce the invasion of water into the film, thus improving the humidity stability of the perovskite. Hence, treatment with MT/IPA during the Cs_2_PbI_2_Cl_2_/CsPbI_2.5_Br_0.5_ crystallization process is helpful when preparing high-quality and stable perovskite solar films.

**Figure 3:**
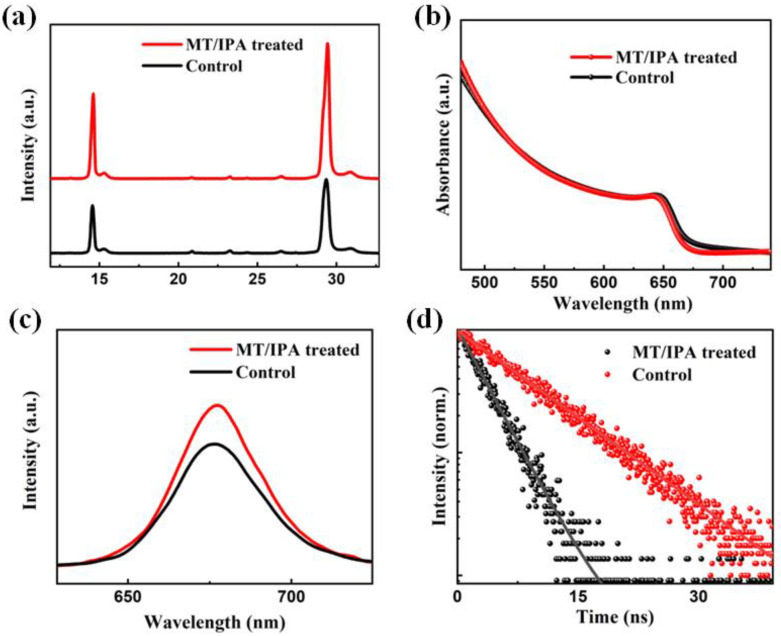
(**a**) XRD spectra of untreated 2D/3D film and MT/IPA-treated 2D/3D film. (**b**) UV-vis absorption spectra of untreated 2D/3D film and MT/IPA-treated 2D/3D film. Spectra of (**c**) PL and (**d**) TRPL for the untreated 2D/3D film and MT/IPA-treated 2D/3D film.

Figure 3a shows the XRD spectra of untreated and MT/IPA-treated films. The diffraction peaks stand at about 14.6° and 29.3° in both of the films, assigned to the (100) and (200) crystal planes of the CsPbI_2.5_Br_0.5_, indicating that the MT/IPA treatment has not changed the composition of the CsPbI_2.5_Br_0.5_. The diffraction peaks of the cubic perovskite CsPbI_2.5_Br_0.5_ of the MT/IPA-treated film are greatly increased compared to those of the untreated film, indicating the MT/IPA treatment enhanced the crystallinity of the perovskite. According to Figure 3b, the UV-vis absorption spectra demonstrate that the untreated film has the same absorption intensity as the MT/IPA-treated film. The absorption edges are both around 680 nm, and the corresponding band gap is 1.82 eV, providing further evidence that the perovskite composition has not changed. Additionally, both of the films have equal absorption intensity. These results show that the MT/IPA treatment has no noticeable effect on the absorption properties of the films.

The effect of MT/IPA treatment on the photoluminescence properties of the films was further investigated. According to Figure 3c, the emission peaks shown in the PL spectra are consistent with the absorption edge results that were previously obtained in the UV-vis absorption spectra. Both the peaks of the untreated and MT/IPA-treated films are at about 680 nm. The MT/IPA-treated film has a higher PL peak than the untreated one, indicating that the perovskite films treated by MT/IPA have fewer defects. Moreover, as shown in Figure 3d, the TRPL was measured to obtain the decay curves of the PL peaks’ intensities. The spectra were fitted using the biexponential function. In addition, the parameters that describe the decay curves are shown in Table 1. τ_1_ represents the rapid attenuation component, which is generally considered to be caused by the fast recombination of the film surface interface [36]. τ_2_ represents the slow attenuation component, which is generally thought to be caused by the recombination of the carriers in the film. The weights of τ_1_ and τ_2_ are given by the values of A_1_ and A_2_, respectively. The weighted average τ_ave_ of τ_1_ and τ_2_ is used to parameterize the carrier lifetime. According to Table 1, the τ_ave_ of the untreated perovskite film is 8.58 ns, while that of MT/IPA-treated films is 18.23 ns. The longer carrier lifetime further confirms that fewer defects exist in the treated films, indicating that the carrier non-radiative recombination of films treated with MT/IPA is effectively suppressed.

**Figure 4:**
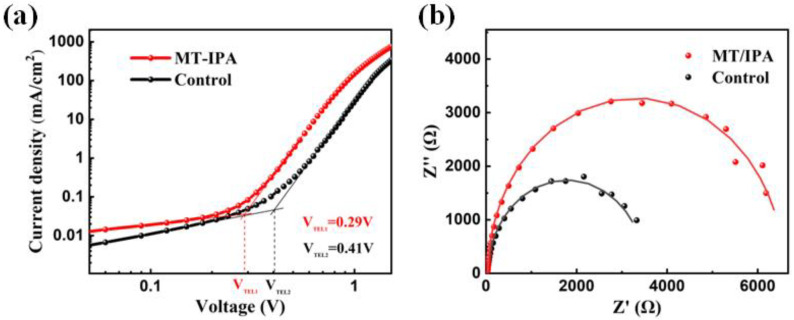
(**a**) SCLC curves of the untreated devices and MT/IPA-treated devices. (**b**) EIS of the untreated devices and MT/IPA-treated devices.

The effect of MT/IPA treatment on carrier recombination in the device was further characterized. As shown in Figure 4a, the dual-electron transport layer devices were tested by SCLC. It was found that the untreated film has a trap-filling limit voltage (*V_TFL_*) of 0.41V, while the *V_TFL_* of the MT/IPA-treated film is reduced to 0.29 V. The following formula demonstrates that the trap state density (*N_t_*) is proportional to the *V_TFL_* [37].

$$N_{t}=\frac{2\varepsilon \varepsilon_{0}V_{TFL}}{eL^{2}}$$

The reduced trap-filling limit voltage indicates a decrease in defects. The detailed result of the calculation is that the *N_t_* of the MT/IPA-treated film (1.32 × 10^16^ cm^−3^) is much lower in comparison to that of the untreated film (1.87 × 10^16^ cm^−3^), indicating that the MT/IPA treatment helps reduce the defects of the film and decreasing the recombination rate. Figure 4b shows the EIS of the untreated and MT/IPA-treated devices. The equivalent circuits were fitted by the EIS Nyquist diagram, and the parameters are shown in Table 2. The untreated device has a series resistance (*R_s_*) of 22.39 Ω, while the *R_s_* of the MT/IPA-treated device is reduced to 15.34 Ω. The recombination resistance (*R_rec_*) of the untreated device is 3480 Ω, and that of the MT/IPA-treated device is 6497 Ω. Lower series resistance and higher recombination resistance mean favorable carrier transport and reduced recombination, which is significant for the improvement of device performance. Besides, the MA/IPA-treated film has a minor bulk defect and a smooth surface morphology, both of which contribute to a reduction in the interface recombination loss that occurs within devices by facilitating hole transportation.

**Figure 5:**
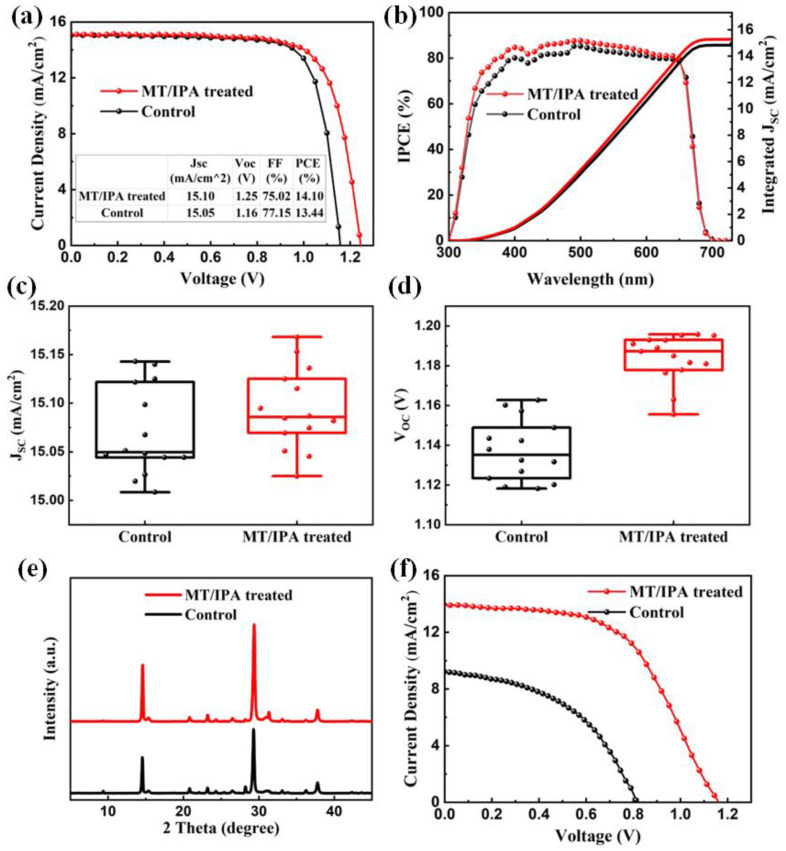
(**a**) *J–V* curves of the untreated and MT/IPA-treated solar cells. (**b**) IPCE curves of the untreated and MT/IPA-treated champion solar cells. The distribution box diagrams of (**c**) *J_sc_* and (**d**) *V_oc_* of the untreated and MT/IPA-treated devices. (**e**) XRD spectra of untreated and MT/IPA-treated perovskite films prepared in the air. (**f**) *J–V* curves of untreated and MT/IPA-treated 2D/3D perovskite cells prepared in the air.

Figure 5a shows the *J–V* curves of the untreated and MT/IPA-treated solar cells, and the corresponding parameters are contained in the attached table. The PCE of the 2D/3D perovskite solar cell without any treatment prepared by the one-step solution method is 13.44%, with an open circuit voltage (*V_oc_*) of 1.16V, short circuit current (*J_sc_*) of 15.05 mA/cm^2^, and filling factor (*FF*) of 77.15%. On the other hand, the improved PCE of the MT/IPA-treated 2D/3D device is 14.10%, with a *V_oc_* of 1.25 V, *J_sc_* of 15.10 mA/cm^2^, and *FF* of 75.02%. The results of the IPCE curves for the untreated and MT/IPA-treated devices are shown in Figure 5b. Both devices have an excellent spectral response in the range of 350 to 650 nm, while the MT/IPA-treated device has a stronger response value, with responsivity of more than 80% in the range of 400 to 600 nm, indicating that the MT/IPA-treated sample has excellent photoelectric conversion efficiency. Integrating the contributions of photons of different wavelengths, the integrated current density of 14.83 mA/cm^2^ for the untreated device and 15.19 mA/cm^2^ for the MT/IPA-treated device are basically consistent with their *J–V* curves. It can be concluded from the distribution box diagrams of *J_sc_* and *V_oc_* of the devices, shown in Figure 5c,d, that the major factor for improving device efficiency is significant increase in *V_oc_*. This is mainly because the MT/IPA-treated devices have fewer defects and good interfacial contact with the perovskite layer and HTL. The long-term stability of the untreated and MT/IPA-treated solar cells in N_2_ is shown in Figure S3 (Supplementary Materials). It can be seen that both devices exhibit excellent stability. In our study, the untreated solar cell retained 83% of the initial PCE, while the MT/IPA-treated solar cell demonstrated better stability, maintaining 87% of the initial PCE after 2400 h. The improved stability should be attributed to the reduced defect density and the densely packed crystals in the MT/IPA-treated film [11].

To better verify the effect of the MT/IPA treatment on devices, the 2D/3D devices without treatment and with MT/IPA treatment were prepared in air with a relative humidity of 10–20%. Figure 5e shows the XRD spectra of the films prepared in the air. Here, the diffraction peaks of the cubic CsPbI_2.5_Br_0.5_ of MT/IPA-treated film are significantly higher than those of the untreated film. As evidenced by the XRD results, the poor crystallinity of the untreated film could have been due to water molecules and impurities in the air that affected the crystallization process. In contrast, the MT/IPA solution could accelerate the evaporation of DMSO, resulting in rapid nucleation and crystallization of perovskite, thus minimizing the influence of air exposure. As a result, the MT/IPA treatment was beneficial in improving the photoelectric performance of devices prepared in the air. The PCE of the devices employed by MT/IPA treatment was increased from 3.52% to 8.91%, as shown in Figure 5f.

## 4. Conclusions

In conclusion, the MT/IPA-treated method was employed to regulate the crystallization kinetics of Cs_2_PbI_2_Cl_2_/CsPbI_2.5_Br_0.5_ perovskite film. The MT/IPA mixed solution has a weak electron donor ability, low boiling point, and poor solubility with halides, which can accelerate the evaporation of DMSO, thus facilitating uniform nucleation and growth within films. By employing the MT/IPA-treated method, the bulk defects and the carrier transmission barrier of the interface were decreased. This was valuable for the reduction of the recombination of the carriers in the device. As a result, the PCE of Cs_2_PbI_2_Cl_2_/CsPbI_2.5_Br_0.5_ solar cells increased from 13.44% to 14.10%. This study proves that the MT/IPA-treated method is beneficial to the preparation of densely packed, smooth, and high-crystallized Cs_2_PbI_2_Cl_2_/CsPbI_2.5_Br_0.5_ perovskite films and can be applied to their preparation in the air.

## Figures and Tables

**Table 1:** Fitting results of photoluminescence attenuation.

	A_1_	τ_1_ (ns)	A_2_	τ_2_ (ns)	τ_ave_ (ns)
Control	0.08	0.59	0.92	9.28	8.58
MT/IPA	0.21	2.41	0.79	22.43	18.23

**Table 2:** EIS corresponding parameters fitted according to Nyquist equivalent circuit.

	*R_s_* (Ω)	*R_tr_* (Ω)	*C_tr_* (F)	*R_rec_* (Ω)	*C_rec_* (F)
Control	22.39	22.93	2.62 × 10^−8^	3480	2.08 × 10^−8^
MT/IPA	15.34	26.43	1.63 × 10^−8^	6497	2.15 × 10^−8^

## Data Availability

The data are available from the authors on request.

## Supplementary Materials

The following supporting information can be downloaded at: https://www.mdpi.com/article/10.3390/nano13071239/s1, Figure S1: UV-vis absorption spectra of unannealed films untreated and treated with different volume ratios of MT/IPA solution; Figure S2: *J-V* curves of solar cells treated with different volume ratios of MT/IPA solutions; Figure S3: Long term stability in N_2_ atmosphere of the untreated and MT/IPA-treated solar cells.
