# Supplementary material for: Highly Efficient 2D/3D Mixed-Dimensional Cs2PbI2Cl2/CsPbI2.5Br0.5 Perovskite Solar Cells Prepared by Methanol/Isopropanol Treatment
Source: Nanomaterials (Basel). 2023 Mar 31;13(7):1239. doi: 10.3390/nano13071239 (PMC10097316; doi:10.3390/nano13071239)
Supplement: Supplementary file 1 [file nanomaterials-13-01239-s001.zip › nanomaterials-2304324-supplementary.pdf]

Article

Highly Efficient 2D/3D Mixed-Dimensional $Cs_2PbI_2Cl_2/CsPbI_{2.5}Br_{0.5}$ Perovskite Solar Cells Prepared by Methanol/Isopropanol Treatment

Bicui Li [1,2], Shujie Yang [1,2], Huifang Han [1,2], Huijing Liu [1,2], Hang Zhao [3], Zhenzhen Li [3], Jia Xu [1,2] and Jianxi Yao [1,2,*]

[1] State Key Laboratory of Alternate Electrical Power System with Renewable Energy Sources, North China Electric Power University, Beijing 102206, China
[2] Beijing Key Laboratory of Energy Safety and Clean Utilization, North China Electric Power University, Beijing 102206, China
[3] College of Metallurgy and Energy, North China University of Science and Technology, Tangshan 063210, China
* Correspondence: jianxiyao@ncepu.edu.cn

Methods for fitting

(a) Time-resolved photoluminescence (TRPL)

The TRPL curves were fitted with a bi-exponential decay function to describe the process of charge extraction.

$$I(t) = A_1e^{(-t/\tau_1)} + A_2e^{(-t/\tau_2)} + \gamma_0 \qquad \text{(S1)}$$

where τ_1 is the rapid attenuation component, which is generally considered to be caused by the fast recombination of the film surface interface, τ_2 is the slow attenuation component, which is generally thought to be caused by the recombination of the carrier in the film. A_1 and A_2 are the weights of τ_1 and τ_2, respectively.

(b) Space charge limited current (SCLC)

The trap density can be calculated from the following Equation (S2):

$$N_t = \frac{2\varepsilon\varepsilon_0 V_{TFL}}{eL^2} \qquad \text{(S2)}$$

where L is the thickness of the perovskite film, ε_r is the relative dielectric constant of perovskite, ε_0 is the vacuum permittivity (8.854×10^{-12} F/m), e is the unit charge with the value of 1.602×10^{-19} C, and N_t is the trap density.

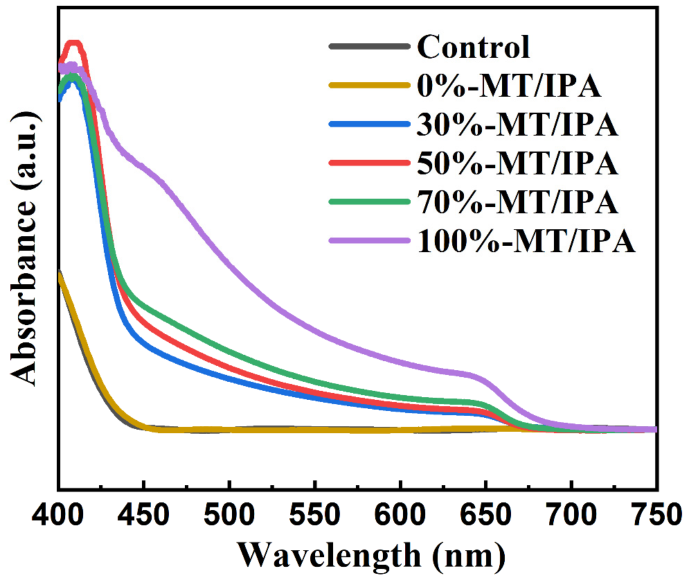

Figure S1. UV-vis absorption spectra of unannealed films untreated and treated with different volume ratios of MT/IPA solution.

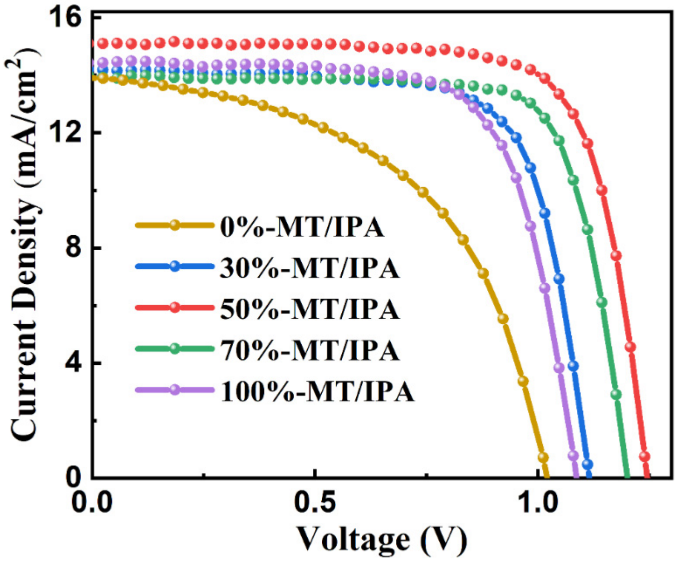

Figure S2. *J*-*V* curves of solar cells treated with different volume ratios of MT/IPA solutions.

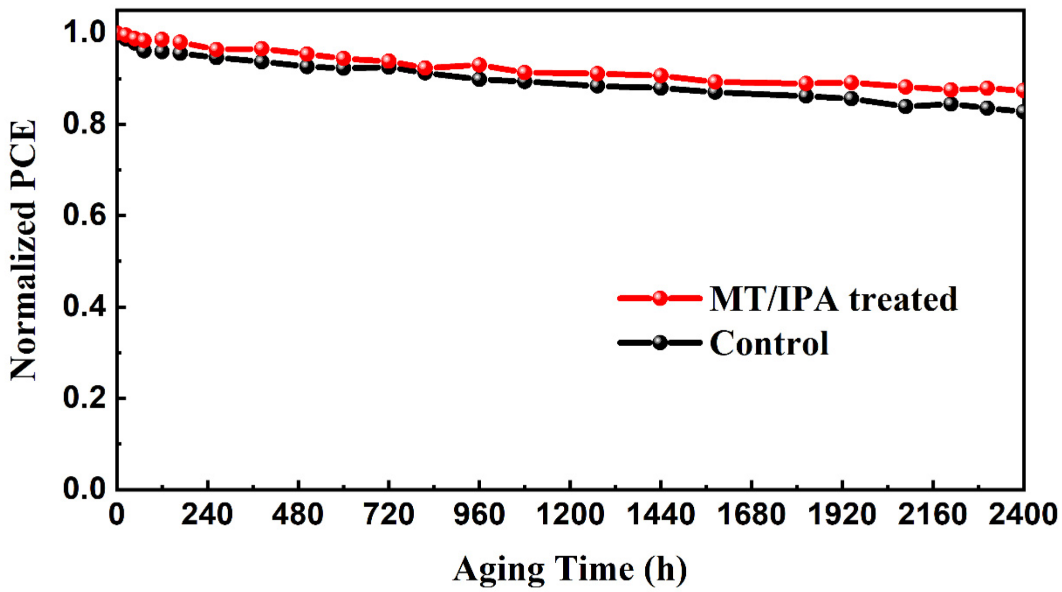

Figure S3. Long term stability in N_2 atmosphere of the untreated and MT/IPA-treated solar cells.
